# On Artificial Production of Rhachitis and Mollities Ossium

**Published:** 1874-01-01

**Authors:** 


### ON ARTIFICIAL PRODUCTION OF RHACHITIS AND MOLLITIES OSSIUM.

FROM A PAPER READ TO THE IMPERIAL SOCIETY OF PHYSICIANS OF VIENNA, OCT. 24, 1873, BY DR. C. HEITZMANN.

*Translated for* THE EXAMINER *from the Allgemeine Wiener Medical Zeitung.*

ENTERING last year on the study of diseases of bones, I met with some statements, while reviewing the literature on the subject, to the effect that lactic acid had been demonstrated in the urine of patients suffering of rickets and mollities ossium, by a number of chemists, and that C. Schmidt had even extracted this substance out of the liquid found in cysts in the bones of such persons. The suggestion, by some authorities, that this acid plays an important part in the ætiology of these affections, induced me to investigate the question by experimentation.

Attempts at artificial softening of bones are not of late origin. In the third decade of this century Guerin raised two packs of dogs, one exclusively on fresh meat, the other on bread and milk. While the latter retained their health, the former, after a slight increase in weight, were attacked with diarrhœa, became emaciated and, finally, rickety; whence the experimenter concluded that rhachitis was dependent on an early weaning and substitution of animal diet.

Some years later Chosset deprived pigeons of all calcareous salts, feeding them only on wheat, with the result of deficient ossification and fragility of the bones. These statements were confirmed by Bibra, who found, besides, that the eggs of chickens, under similar treatment, did not possess a calcareous shell, and that at last the oviparous process ceased altogether.

Within the last year Wegener has observed that a typical rhachitis can be produced in chickens by the deprivation of calcareous salts simultaneous with the administration of phosphorous.

My own experiments were commenced on the 9th of April, a year ago, on a large young hound, who received daily two hypodermic injections of dilute lactic acid, and, besides, had his food flavored with the same article. His bill of fare was limited to milk, boiled meat, white bread, cooked potatoes, and fat; while bones and rye-bread were excluded on account of their richness in calcic salts. A single week sufficed to produce marked appearances, among the first of which were convulsions during the sleep of the animal, proceeding from the feet to the muscles of the back, sometimes so violent as to awaken the animal. This was soon followed by diarrhœa, the dog became dejected and exhausted by the least exertion, restless, and emaciated. A fortnight later, swelling of the epiphyses of the fore feet was observed, and, soon after, the same in the hind feet, while tumefaction occurred at the connection of the ribs with the cartilages, and the limbs began to curve. This curvature increased rapidly, reaching its climax between the fifth and sixth week of the use of the drug; the bones of the extremities were arched, with the convexity outward, the forefeet even S-shaped; the hound was metamorphosed into a terrier.

Such a striking result encouraged me to further experiments, which have since been performed on five dogs, seven cats, two rabbits, and one squirrel. Two of the dogs were lost by peritonitis, probably from too concentrated, and an excess of, the lactic acid. The other animals have been observed during a period varying between sixteen days and thirteen months. I soon learned that injection of the agent was unnecessary.

The dogs and cats, young animals in the process of development, suffered, in the second week of the experiment, of a tumefaction of the epiphyses of the long bones and the connections of the ribs and cartilages, augmenting in size till the fifth or sixth week, with curvature of the extremities, catarrhal inflammation of the conjunctival, bronchial, gastric, and enteritic mucous lining, and convulsions.

Though these symptoms presented by the carniverous animals are characteristic of rhachitis, the stern demands of science called for a positive proof that this was the actual disease, which, however, was really given by a subsequent microscopical comparison of the bones of the animals with those of rickety children.

These appearances remained, then, stationary up to the sixth or eighth week, when, though the lactic acid alimentation was continued, they began to decline, and finally disappeared to some degree. Notwithstanding my discouragement at this unexpected result, the course of experimentation was not interrupted, and my patience was at last rewarded with success, for, as the influence of the acid was kept up, a repetition of the catarrhal symptoms was observed, the growth was retarded, and emaciation the constant condition.

After four or five months, desiring to investigate especially osseous inflammations, I selected for this purpose several of the dogs and cats, with the intention of producing fractures of the long bones in some, of the scapulæ in others; but, while attempting to fracture the thigh of a narcotized dog over the edge of the table, I was surprised to find the limb as flexible as whalebone, and that very little exertion sufficed to complete the process, which, in healthy animals, requires considerable force. The dissection showed a marked attenuation of the cortical laminæ in long bones, and a parchment-like thinness and flexibility of the scapula, while the marrow appeared highly vascular, and, in a section steeped in chromic-acid solution, expanded above and beyond its enclosing walls.

In the hound previously mentioned, killed after eleven months experimentation, on suspicion of hydrophobia, the cortical laminæ of the femur, of two or three mm. thickness in corresponding healthy animals, was reduced to one mm., and at some spots where the enlarged marrow had caused erosion, to five-tenths mm. The diagnosis of mollities ossium, so appropriate in this case, was confirmed by the microscopic examination of the osseous tissue of two patients dead with the disease, the peculiar appearances of which were similar.

The symptoms presented by the herbiverous animals experimented upon were somewhat different, more of them having suffered of swelling of epiphyses or curvature of the extremities. The two rabbits, both of the age of five months, died after three and five months, respectively, apparently from inanition, though they had been well fed on vegetables, bread, and boiled potatoes, and had evidently been growing. In the *post-mortem* examination, the long bones showed a rather thin, compact tissue, and remarkably brittle, but the microscopic appearances were not sufficiently characteristic of the lesions of mollities ossium to justify such a diagnosis. In the meantime the squirrel died, at the age of three years, under circumstances referable only to old age; but as I am aware, from previous experience, that the duration of life in these animals reaches five years, it seems to me probable that the lactic acid administered, *guttatim*, for thirteen months was the means of hastening somewhat the animal's death. A dissection, one hour after death, showed the long bones light, soft, flexible, and apt to break without snapping; the ribs delicate and thin; and the cranial bones and scapula of the thickness and flexibility of paper; in fact, the complete picture of mollities ossium.

The direct inferences of my experiments are, therefore, that, in carnivorous animals, rhachitis, and, subsequently, mollities ossium, are produced by the continued administration of lactic acid; while, in herbiverous ones, the latter affection, not preceded by the former, is the result; and thus the hypothesis that rhachitis, the disease of youth, and mollities, the affection of middle-age, are identical morbid processes, is well sustained, since the same agent produces either, according to the age of the animal.

Still, many points of interest are involved in uncertainty. The chemist can trace the conversion of hydrocarbons into lactose, and this into lactic acid; but why this pathological accumulation of a normal, proximate principle? No doubt, however, the department of ætiology has received an addition, though my present paper is limited to the contents of some statements of mine published by the Imperial Academy of Sciences, in June last

I may, finally, remark that I am at present placing a couple of cats (male and female) on the use of lactic acid, with the hope of perpetuating the disease.

At the close of this report the author exhibited a fœtus of seven and one-half months, the result of a miscarriage, during the previous night, of the woman who had attended to the feeding and administration of the acids to the animals; which child, dying after several respirations, presented marked appearances of congenital rickets; but that this occurrence was the result of the occupation of the mother, the author was not prepared to affirm.

